# Lebensstil: körperliche Aktivität und Training in der Prävention und Therapie des Typ 2 Diabetes mellitus (Update 2023)

**DOI:** 10.1007/s00508-023-02187-3

**Published:** 2023-04-20

**Authors:** Claudia Francesconi, Josef Niebauer, Paul Haber, Othmar Moser, Raimund Weitgasser, Christian Lackinger

**Affiliations:** 1 Sonderkrankenanstalt Rehabilitationszentrum Alland, Alland, Österreich; 2 Universitätsinstitut für Präventive und Rehabilitative Sportmedizin, Landeskrankenhaus Salzburg – Universitätsklinikum, Paracelsus Medizinische Privatuniversität, Salzburg, Österreich; 3 Universitätsklinik für Innere Medizin II, Medizinische Universität Wien, Wien, Österreich; 4 Klinische Abteilung für Endokrinologie und Stoffwechsel, Universitätsklinik für , Innere Medizin, Medizinische Universität Graz, Graz, Österreich; 5 Institut für Sportwissenschaft, Universität Bayreuth, Bayreuth, Deutschland; 6 Abteilung für Innere Medizin, Privatklinik Wehrle-Diakonissen, Salzburg, Österreich; 7 Österreichische Gesellschaft für Public Health, Wien, Österreich

**Keywords:** Körperliche Aktivität, Inaktivität, Standardisierte Bewegungsprogramme, Kardiaovaskuläres Risiko, Bewegungberatung, Health enhancing physical activity, Standardised exercise programme Cardovasculär risk, Physical activity counselling

## Abstract

Lebensstil, insbesondere regelmäßige körperliche Aktivität, ist ein wichtiger Bestandteil in der Prävention und Therapie des Typ 2 Diabetes mellitus und sollte fester Bestandteil jeglicher Betreuung von Patient:innen sein. Es besteht breiter Konsens, dass eine effiziente Diabetes-Prävention und Therapie in den meisten Fällen auch von einer Modifikation des Lebensstils begleitet sein muss.

Ziele der Förderung der körperlichen Aktivität sind zunächst das Training des Herz-Kreislaufsystems, Kräftigung der Muskulatur, Steigerung des Energieumsatzes und die Reduktion von Inaktivität. Für einen substanziellen gesundheitlichen Nutzen sind wöchentlich mindestens 150 min aerobe körperliche Aktivität mit mittlerer oder höherer Intensität und zusätzlich muskelkräftigende Bewegungen erforderlich.

Das Ausmaß des positive Effektes von Bewegung steht in direktem Verhältnis zum Grad der erreichten kardiorespiratorischen Fitness, und kann nur durch entsprechendes Training aufrechterhalten werden. Körperliches Training ist in jedem Alter für beide Geschlechter wirksam und effektiv. Durch die Reduktion der Insulinresistenz und funktionelle Verbesserung der Insulinsekretion hat körperliches Training positiven Einfluss auf die Glykämie und zusätzlich wird das kardiovaskuläre Risiko gesenkt.

Im Speziellen hat Training nicht nur positiven Einfluss auf die Glykämie durch Verbesserung der Insulinresistenz und funktionelle Verbesserung der Insulinsekretion zu nehmen, sondern ist auch in der Lage, das kardiovaskuläre Risiko zu senken.

Inaktivität per se gilt unabhängig vom Konstrukt der körperlichen Aktivität als Risikofaktor. Insbesondere langandauernde sitzende Tätigkeit soll vermieden werden.

Standardisierte, regionale und angeleitete Bewegungsprogramme sind bestens geeignet, um ein ausreichendes wöchentliches Ausmaß an gesundheitsfördernder körperlicher Aktivität zu erreichen. Zusätzlich fordert die Österreichische Diabetes Gesellschaft die Position der Bewegungsberater:in als fixen Bestandteil eines multidisziplinären Behandlungsansatzes. Leider gab es in den letzten Jahren weder im Aufbau standardisierter Bewegungsangebote noch in der Bewegungsberatung erfolgsversprechende Entwicklungen.

## Abstract (en)

Lifestyle, in general and particularly regular physical activity, is known to be an important component in the prevention and therapy of type 2 diabetes.

To gain substantial health benefits, a minimum of 150 min of moderate or vigorous intense aerobic physical activity and muscle strengthening activities per week should be performed. Additionally, inactivity should be recognized as health hazard and prolonged episodes of sitting should be avoided.

Especially exercise is not only efficient in improving glycaemia by lowering insulin resistance and enhance insulin secretion, but to reduce cardiovascular risk. The positive effect of training correlates directly with the amount of fitness gained and lasts only as long as the fitness level is sustained. Exercise training is effective in all age groups and for all genders. It is reversible and reproducible.

Standardized, regional and supervised exercise classes are well known to be attractive for adults to reach a sufficient level of health enhancing physical activity. Additionally, based on the large evidence of exercise referral and prescription, the Austrian Diabetes Associations aims to implement the position of a “physical activity adviser” in multi-professional diabetes care. Unfortunately, the implementation of booth—local exercise classes and advisers is missing so far

## Grundlagen und Nutzen

Regelmäßige Bewegung und damit verbunden eine Verbesserung der körperlichen Leistungsfähigkeit ist für alle Menschen gesundheitswirksam. Im Besonderen profitieren jedoch Menschen mit metabolischem Syndrom bzw. Typ 2 Diabetes mellitus (T2DM) von Bewegung. Körperliche Aktivität bildet die Grundlage jeder Therapie und ist nicht nur eine Ergänzung der medikamentösen Maßnahmen. Die Ursache liegt in der Erkrankung zugrunde liegenden Insulinresistenz, welche sowohl durch Ausdauer- als auch durch Krafttraining grundlegend beeinflusst werden kann [1–7].

Durch Ausdauertraining kommt es zur effizienteren Aufnahme und Verstoffwechselung von Glukose in der Muskelzelle. Da die Muskulatur 50–70 % der aufgenommenen Glukose verbraucht, ist eine weitgehende Normalisierung des Glukosestoffwechsels in der Muskelzelle essentiell für eine Verbesserung der Insulinresistenz insgesamt [8, 9]. Krafttraining kann über einen zusätzlichen Glukosetransporter die Glukoseaufnahme in die Zelle verbessern und bewirkt durch Zunahme der Muskelmasse vor allem eine Bedarfserhöhung und eine Steigerung des Grundumsatzes, verbunden mit einer positiven Beeinflussung der Gewichtsentwicklung (Gewichtsreduktion), was vor allem bei zumeist sarkopenen, adipösen Stoffwechselpatient:innen von Vorteil ist. Zusätzlich kommt es durch die erhöhte Muskelmasse zu besserer Gelenks- und Wirbelsäulenstabilität, verringerter Morbidität betreffend Stürze und Folgeschäden sowie positiver Beeinflussung von Osteoporose und deren Folgen [10, 11]. Insbesondere bei älteren und kardiorespiratorisch eingeschränkten Individuen ist Krafttraining auf Grund des geringeren Trainingsumfanges und Aufwandes oft einfacher einzusetzen, sollte aber wenn immer möglich durch Ausdauertraining ergänzt werden [12, 13].

## Prävention des T2DM

Für einen substantiellen gesundheitlichen Nutzen sollten erwachsene Frauen und Männer wöchentlich mindestens 150 min aerobe Aktivität mit mindestens mittlerer Intensität erreichen (oder 75 min mit höherer Intensität bzw. eine äquivalente Kombination aus beiden) und zusätzlich muskelkräftigende Übungen durchführen [14]. Für einen weitreichenden gesundheitlichen Nutzen wäre das doppelte Ausmaß erforderlich. Mittlere Intensität ist definiert mit einem Energieverbrauch von 3–6 METs [15]. Naturgemäß ist jede Bewegung besser als keine Bewegung, aber die gesundheitlichen Effekte von körperlicher Aktivität mit leichter Intensität sind deutlich geringer als jene mit mittlerer oder hoher Intensität [16]. Laut den nationalen Bewegungsempfehlungen ist der Wechsel von körperlich inaktiv zu ein wenig aktiv ein erster, wichtiger Schritt. Auch die aktuellen Leitlinien der American Diabetes Associaton thematisieren erstmalig, dass Alltagsaktivität wichtig ist – vorrangig um Perioden mit Inaktivität zu unterbrechen. Wichtig ist jedoch die Unterscheidung, dass körperliches Training eine spezifische Form der körperlichen Aktivität ist, mit dem klaren Ziel, die Fitness und Leistungsfähigkeit zu verbessern. Dennoch sind sowohl die Alltagsaktivität als auch das körperliche Training wichtig in der Prävention und Therapie des Diabetes Mellitus Typ2.

Gegenwärtig gibt es in Österreich nur wenige Studien, welche das Erreichen der Bewegungsempfehlungen untersuchte: bei der körperlich aktivsten Altersgruppe der österreichischen Allgemeinbevölkerung, den 20- bis 29-Jährigen, erreichen lediglich 39,4 % die Bewegungsempfehlungen. Selbst bei Medizinstudent:innen erreicht diese nur eine Minderheit [17]. Im Euro Heart Survey wurde gar gezeigt, dass 86 % der männlichen und 94 % der weiblichen Patient:innen die Empfehlungen für körperliche Aktivität in den Leitlinien nicht erfüllten [18]. Dabei ist seit Jahren bekannt, dass gezielte körperliche Aktivität besser geeignet ist, um die Zahl an Diabetes-Neuerkrankungen zu reduzieren als die herkömmliche Medikation ([19–21]; Tab. 1).

**Table 1:** 

*Aerobe Aktivität*	
Umfang mit mittlerer Intensität	≥ 150 min pro Woche
Oder Umfang mit höherer Intensität Regelmäßigkeit	≥ 75 min pro Woche ≥ 3 × pro Woche
*Muskelkräftigende Aktivität*	
Regelmäßigkeit	≥ 2 × pro Woche
Intensität	Hypertrophie- oder Kraftausdauertraining
Umfang	9 Muskelgruppen mit jeweils 4 Sätzen pro Woche
*Alltagsaktivität*	
Aktivitäten mit leichter Intensität	Jegliche Aktivität, auch mit leichter Intensität ist zu fördern
Inaktivität	Jede Bewegung ist besser als keine. Regelmäßige körperliche Aktivität muss in den Alltag integriert werden

Die Empfehlungen für Umfang und Intensität von körperlicher Aktivität von Seiten der Österreichischen Diabetes Gesellschaft sind in Analogie zu den Empfehlungen internationaler Fachgesellschaften [23–28] (ADA level A, CDA Grade B level 2, NVL A): siehe Tab. 1. Diese sind auch ident mit den aktuellen nationalen Bewegungsempfehlungen für Erwachsene – mit oder ohne chronische Einschränkungen. Diese schließen auch explizit Menschen mit Diabetes Mellitus Typ2 mit ein [16]. Sollten Personen auf Grund ihrer chronischen Erkrankung die Empfehlungen nicht umsetzten können, sollten sie dennoch soweit wie möglich körperlich aktiv sein und Inaktivität vermeiden.

## Therapie des T2DM

Die Art, Dauer, Intensität und der wöchentliche Umfang an körperlicher Aktivität in der Therapie unterscheidet sich per se nicht von den Bewegungsempfehlungen in der Prävention.

Ältere oder behinderte Menschen sollen nach Maßgabe ihrer Möglichkeiten ebenfalls die obigen Ziele anstreben und zusätzlich ein Gleichgewichts- und Flexibilitätstraining durchführen.

Die gezielte Beratung, das Erarbeiten persönlicher Zielsetzungen, das Protokollieren der Aktivitäten, Kontrolle und Besprechung der Protokolle sowie kontinuierliche Motivation dienen dem niederschwelligen Zugang und der Überwindung von Widerständen von Seiten der Patient:innen (CDA Grade B Level 2). Jedenfalls soll eine möglichst genaue Bewegungsanleitung mit den Patient:innen besprochen werden, um die Umsetzung zu erleichtern [29, 30]. Dazu gehört:

- die Auswahl der geeigneten Bewegungsform,
- die Dauer der Belastung,
- die Intensität der Belastung,
- die Anzahl der wöchentlichen Belastungen (Frequenz).

## Einschränkungen

Es gibt keine Kontraindikation für Bewegungstherapie bei Menschen mit Typ 2 Diabetes mellitus, jedoch muss auf bestehende Komorbiditäten Rücksicht genommen werden, um Schäden zu vermeiden [31, 32]. Diabetische makro-/mikrovaskuläre Komplikationen, die eine spezielle Abklärung bzw. Aufklärung der Patient:innen notwendig machen, sind:

- Proliferative Retinopathie (cave: Blutdruckspitzen: Kraftausdauer- statt Hypertrophietraining; moderates statt intensives Ausdauertraining),
- Periphere Diabetische Neuropathie (cave: Druckstellen und Charcotfrakturen), autonome Neuropathie,
- Klinisch symptomatische koronare Herzerkrankung/Herzinsuffizienz (Ergometrie, Herzfrequenzgesteuertes Training),
- Möglichkeit der Hypoglykämie: bei Therapie mit Insulinsekretagoga und/oder Insulin muss die Patient:in über die Möglichkeit einer durch Bewegung ausgelösten Unterzuckerung aufgeklärt und die entsprechenden Gegenmaßnahmen besprochen werden (ADA ohne Angabe von Evidenzgrad, CDA Grad D + Consensus).

Vorübergehende Kontraindikationen sind alle akuten Erkrankungen, z. B. fieberhafte Infekte.

Routinemäßige Belastungsuntersuchungen bei Menschen mit Typ 2 Diabetes mellitus ohne koronare Herzkrankheit werden nicht empfohlen [33]. Ein Bewegungsprogramm mit leichter oder mittlerer Intensität kann bei asymptomatischen Patient:innen mit normalem Ruhe-EKG und ohne bekannter kardiovaskulärer Begleiterkrankung auch ohne vorheriger Belastungsuntersuchung umgesetzt werden [1]. Selbstverständlich ist eine genaue Anamnese und Erhebung von kardiovaskulären Risikofaktoren unerlässlich in der Betreuung von Menschen mit T2DM. Auch wenn eine routinemäßige Belastungsuntersuchung nicht empfohlen wird, ist dennoch für eine effiziente und risikoarme Trainingssteuerung, der Dokumentation des Trainingserfolgs, sowie zur Objektivierung der wichtigsten kardiovaskulären Risikofaktoren eine symptomlimitierte Ergometrie unabdingbar [34].

## Inaktivität

Inaktivität wurde lange Zeit als das untere Ende eines Aktivität-Kontinuums gesehen, jedoch ist diese Betrachtung nicht mehr zeitgemäß [35, 36]. Das Ausmaß der Inaktivität beträgt in Europa bereits mehr als 40 h pro Woche [37]. Moderne Büroarbeit wird zum überwiegenden Anteil im Sitzen verbracht und steht somit kausal im Zusammenhang mit Inaktivität. Demnach wird 77 % der Büroarbeitszeit körperlich inaktiv verbracht [38]. Generell können auch Personen, welche die Kriterien der Bewegungsempfehlungen erreichen, durch ununterbrochene sitzende Tätigkeit einem gesundheitlichen Risiko ausgesetzt sein. Das systematische Unterbrechen von sitzender Tätigkeit nach idealerweise 30 min bringt bereits sowohl kurzfristige Effekte, einen gesteigerten Blutfluss und beeinflusst die Glukosekonzentration, als auch langfristig werden Risikofaktoren für kardiovaskuläre Erkrankungen, sowie Diabetes mellitus assoziierte Morbidität und Mortalität reduziert [39–42]. Die nationalen Bewegungsempfehlungen beschreiben, dass langandauerndes Sitzen überhaupt vermieden, und immer wieder durch Bewegung unterbrochen werden sollte [16]. Folglich sollten der Arbeitsalltag, der Arbeitsweg und die Freizeit möglichst viel körperliche Aktivität und körperliches Training beinhalten.

## Schnittstellenmanagement als eine zentrale Herausforderung

Internationale Leitlinien haben bereits erkannt, dass die zukünftigen Herausforderungen im Bereich der Förderung der körperlichen Aktivität darin liegen, dass Zugänge zu geeigneten regionalen Einrichtungen, welche standardisierte Bewegungsprogramme umsetzen können, gefunden und gefestigt werden müssen [43]. Sportvereine spielen hier eine wichtige Rolle, und es konnte gezeigt werden, dass standardisierte Sportvereinsprogramme nicht nur das Ausmaß der körperlichen Aktivität erhöhen, sondern auch die Fitness signifikant verbessern [44]. Die Zusammenarbeit zwischen dem Gesundheitssystem und regionalen Sportvereinen hat somit durchwegs Potenzial, das aber noch nicht Ansatzweise zu einer flächendeckenden Versorgung mit Bewegungsprogrammen geführt hat [45]. Selbst in den wenigen Fällen, wo geeignete, zielgruppenspezifische Bewegungsprogramme vorhanden sind, gibt es viele Barrieren in der Empfehlung dieser Programme [46]. Als häufige Barrieren werden oft genannt: Zeitmangel (92 %), fehlende Standardisierung des Schnittstellenmanagements (88 %) oder geringe Patient:innenencompliance (32 %) [47]. Jedoch wurde bereits gezeigt, dass knapp 50 % der Patient:innen in einer Diabetes-Ambulanz Interesse an einem zielgruppenspezifischen Bewegungsprogramm haben.

Immerhin knapp ¼ aller Patient:innen hat in weiterer Folge ein Bewegungsprogramm aktiv in Anspruch genommen [44].

Als Lösungsansatz fordert die ÖDG die Position der Bewegungsberater:in zu etablieren und beruft sich auf eine breite Evidenz betreffend „exercise referral“ und „exercise prescription“ [48–53]. Das ÖDG-Positionspapier zur Bewegungsberat:in basiert auf dieser breiten Evidenz und leitet daraus folgende Handlungsfelder ab:

- Bewegungsanamnese
- Ist-Analyse
- Intervention (Beratung, Mustertraining, Risikomanagement, soziale Unterstützung und Bewältigungsstrategien, Information über regionale Bewegungsangebote)
- Zielvereinbarung und Kontrolle

Nach einer erfolgreichen Erstberatung sind quartalsweise Folgeberatungen geplant.

Das Ziel der ÖDG für die nächsten Jahre ist die dauerhafte Implementierung der Bewegungsberatung im ambulanten und niedergelassenen Bereich (http://www.oedg.org/pdf/1410_Positionspapier_Bewegungsberater.pdf) (Tab. 2).

**Table 2:** 

		Erstberatung	Folgeberatungen
Krankenakte	Lesen und notwendige Information verstehen	✓	✓
Bewegungsanamnese	Aktuelle körperliche Aktivität	✓	✓
	Gezieltes Training	✓	✓
	Bevorzugte Bewegungsform aktuell	✓	–
	Bevorzugte Bewegungsform in der Vergangenheit	✓	–
Ist-Zustand	Bestimmung der körperlichen Fitness	✓	✓
	Veränderungen seit der letzten Beratung	–	✓
Intervention	Aktuelle körperliche Aktivität	✓	✓
	Selbstständiges Training		
	Angeleitetes Training		
	Kombinationen		
Mustertraining	Krafttrainingsübungen	Bei Bedarf	Bei Bedarf
	Intensitätssteuerung Ausdauer	Bei Bedarf	Bei Bedarf
Risikomanagement		✓	✓
Information über regionale Angebote		✓	✓
Soziale Unterstützung und Bewältigungsstrategien		✓	✓
